# The Morbidity and Mortality Conference: A Century-Old Practice with Ongoing Potential for Future Improvement

**DOI:** 10.1055/s-0043-1760836

**Published:** 2023-01-31

**Authors:** Marit S. de Vos, Merel J. Verhagen, Jaap F. Hamming

**Affiliations:** 1 Directorate of Quality and Patient Safety, Leiden University Medical Center, Rotterdam, the Netherlands; 2 Department of Surgery, Leiden University Medical Center, Leiden, the Netherlands

**Keywords:** morbidity and mortality conferences, team meetings, patient safety, quality improvement, continuing education

## Abstract

**Objective** To discuss practical strategies to consider for morbidity and mortality conferences (M&M).

**Materials and Methods** This article reflects on (i) insights that can be drawn from the M&M literature, (ii) practical aspects to consider when organizing M&M, and (iii) possible future directions for development for this long-standing practice for routine reflection.

**Results** M&M offers the opportunity to learn from past cases in order to improve the care delivered to future patients, thereby serving both educational and quality improvement purposes. For departments seeking to implement or improve local M&M practice, it is difficult that a golden standard or best practice for M&M is nonexistent. This is partly because comparative research on different formats is hampered by the lack of objective outcome measures to evaluate the effectiveness of M&M. Common practical suggestions include the use of (i) a skillful and active moderator; (ii) structured formats for case presentation and discussion; and (iii) a dedicated committee to guide improvement plans that ensue from the meeting. M&M practice is affected by various sociological factors, for which qualitative research methods seem most suitable, but in the M&M literature these are sparsely used. Moreover, aspects influencing an open and blame-free atmosphere underline how local teams should tailor the format to best fit the local context and culture.

**Conclusion** This article presents practice guidance on how to organize and carry out M&M This practice for routine reflection needs to be tailored to the local setting, with attention for various sociological factors that are at play.

## Introduction

The practice of morbidity and mortality conferences (M&M) emerged in the early 20th century, when the first medical specialties, particularly general surgery and anesthesiology started to openly discuss complicated cases periodically. 1 One of the pioneers of systematic efforts to reflect on outcomes in healthcare was the American surgeon Ernest Codman, who monitored morbidity and mortality rates in his institution in the early 1900s. 1 Currently, M&M has become a standard practice for many medical specialties to learn from adverse outcomes, and is mandated by many residency programs as part of specialty training. 2 3 The conference has also famously been referred to as “the golden hour of the surgical workweek.” 4 In addition to their evident educational purpose, these meetings also have come to serve a function for quality and safety improvement. 5 6 7 As such, modern M&M directs participants' attention not only outward, toward “evidence” as a source of knowledge, but also inward, toward opportunities for “systems improvement.” 7 However, there is no definitive evidence that the conferences are actually effective for achieving the educational and improvement objectives. 8 9 10 Generating evidence of effectiveness is challenged by the lack of objective outcome measures for M&M, due to the inability to single out the meeting's effect on generic clinical outcomes. More generally, assessing the value of M&M practice is hampered by the large practice variation that exists. Some departments infrequently organize M&M to discuss an interesting case, whereas others systematically reflect on local and benchmarking data in a more structured manner. This practice variation is partly due to the lack of a best practice to offer guidance on how these meetings should best be organized. 9 11 12 This article will reflect on (i) insights that can be drawn from the M&M literature, (ii) practical aspects to consider when organizing M&M, and (iii) possible future directions for the development of this long-standing practice for routine reflection.

## Insights from M&M Literature: Structural and Cultural Factors

The practice of M&M conference has been the focus for various types of studies. Most research focuses on evaluating current M&M practice 13 14 15 16 or testing interventions to optimize the conference's format, such as implementing a structured format for preparation. Assessing the value of M&M, in terms of its output and effectiveness, is challenged by the lack of an instrument to evaluate this objectively and comprehensively. Clinical outcomes, such as complication rates, are affected by many different other factors, and it is not possible to isolate the conference's impact from other advancements in clinical practice. Our group recently published a scoping review of the literature that portrayed a large heterogeneity in outcomes measures used to evaluate M&M effectiveness; with the most commonly used categories relating to “conference characteristics,” “participant experiences,” “medical knowledge or competencies,” “actions for improvement”, and “clinical outcomes.” 6 Most studies appeared to use quantitative approaches, mostly surveys. In contrast to surveys pointing out the extent of agreement with predetermined topics, qualitative approaches using interviews or observations may help to develop a deeper understanding of a phenomenon of interest, such as M&M practice and the many complex mechanisms that influence it (e.g., local culture and context). It seems most sensible to first clearly determine the objectives of M&M practice and then select a combination of suited outcome metrics, both quantitative and qualitative. 6

Greater use of qualitative methods in M&M research could, for example, be deployed to help appreciate the many sociological benefits of the conference and help to understand all factors that make discussing complicated cases at M&M such a delicate matter. The sociologist Charles Bosk famously describes M&M as “putting on the hair shirt of professional responsibility” in his landmark study on surgical training. 17 Ethnographic research has described how staff are teaching various types of skills, attitudes, and behaviors through their role-modelling at M&M conferences, such as tolerating ambiguity and dealing with the emotional impact of complicated cases. 18 A qualitative assessment of barriers and facilitators for successful M&M practice, illustrated how complex factors such as hierarchy and team spirit, can both hurt and help in some respects. 19 To illustrate, a strong team spirit was considered a positive factor (e.g., generating mutual support), but at the same time a potential barrier as one may withhold certain comments or critique to avoid the risk of potentially offending another team member. In short, this study pointed to the importance of making sure M&M attendees are motivated to participate and take action, well-informed to identify targets and plans for improvement, and willing and able to realize plans that ensue from the meeting. More specific success factors highlighted in this surgical study, which were also found in studies in other medical settings, include the importance of resources (i.e., time and staff), 20 21 leadership buy-in and presence, 21 22 input from all staff levels, 20 21 22 23 and loop closure. 20 22 In the wider literature on organizational learning and implementation science, the importance of individual and team factors is frequently stressed since, after all, “culture eats strategy for breakfast.” 24 25 26

Even though the literature commonly highlights the need for an atmosphere free of “shame and blame” at the conference, 13 27 28 29 30 it remains poorly understood how exactly to establish or support this. Moreover, having an open and blame-free environment might be considered a precondition for successful M&M, but one could also argue that it is a consequence of good M&M practice, which will help build this desired atmosphere and culture. Yet, even in the most supportive atmosphere, feelings of shame and guilt will inherently be present as clinicians involved in a complicated case will be affected by the “second victim” phenomenon, frequently accompanied by self-blame. 31 In terms of research domains, the desired “open and blame-free environment” can be linked to “psychological safety,” and this research field can provide leads on how to foster an environment in which people feel comfortable with speaking up with questions, observations and concerns, even if those may be perceived as difficult or “bad news.” 32 The construct of psychological safety is described as an individual's perception of the consequences of taking interpersonal risks, such as acknowledging shortcoming of competence, asking for help or trying something new. 33 These perceived consequences will determine how one will respond in everyday clinical practice, as this is filled with situations with uncertainty, or a need for creativity, assistance, or collaboration. Imaginably, the extent of psychological safety is affected by discussions taking place at M&M conferences, during which interpersonal risks such as voicing concerns, asking questions, and giving feedback are common. Therefore, one may argue that “psychological safety” is needed, but also shaped, during M&M, which warrants more research into this reciprocal relationship.

## Practical Guidance for Setting Up and Improving M&M Conferences

For those aiming to develop or update their M&M conference, the literature offers various suggestions. When implemented well, various strategies seem promising, such as improving case selection by using an electronic registry or standardizing presentation formats. 34 A Canadian group has developed, implemented, and tested a comprehensive model for M&M, focusing on case selection and structured analysis, interprofessional and multidisciplinary involvement, the use of a facilitator, summarizing and disseminating lessons learned, and creating an administrative pathway for issues identified at M&M to be brought forward for action by a quality committee. 30 35 However, many of the studies on M&M omit to provide a comprehensive description of all relevant practical aspects, including the conference's goals (e.g., educational, quality improvement, or a combination), structure (e.g., participants, details on frequency or duration), and process or content (e.g., selection of cases), limiting the conceivable implications for others. 11 Moreover, one needs to tailor the format to local characteristics, such as case volume or whether team members are co-located or distributed. While it remains difficult to establish a single evidence-based design for successful M&M, the literature still provides a rich source of information on various local experiences. Drawing on various studies on M&M practice from our group 6 16 19 36 37 as well as others, 1 5 38 39 some practical guidance on how to organize M&M is outlined in Table 1 .

**Table 1:** Practical guidance on how to organize and carry out M&M a

Goals	
Collectively determine the goals and primary focus (e.g., education, identifying, and addressing quality/safety issues or both), and consider explicitly stating these at the start of M&M as well as explicitly framing the purpose as non-blaming	
**Structure**	
Frequency	Periodic (e.g., weekly, bi-weekly, or monthly) on the same day/time
Location	Preferably near the clinical workplace (e.g., increases attendance rates). Consider allowing hybrid participation for remote attendees
Duration	Mostly 60–90 minutes (trade-off between thoroughness and feasibility)
Planning	Dedicate time and staff to M&M and ensuing plans for improvement. Make sure no elective interventions, outpatient clinic appointments or other activities are planned at the same time
Participants	Determine who should/may attend, and consider a smaller, focused setting such as that of the subspecialty team rather than entire department. Consider (occasionally) inviting other disciplines (e.g., nurses) or specialties (benefits: more perspectives/information, helps implementing actions; risks: “outsiders” may hamper openness). Also consider (forms of) patient participation 38 39
Attendance	Decide whether attendance is encouraged or mandated (e.g., using sign-in sheets). Providing food and beverages may help
Presenters	Determine who should present, in terms of seniority and involvement in the case. Having residents present may create learning experiences but may also create a vulnerable situation due to dependencies and power dynamics. Presenters who were involved in cases have more accurate and detailed information, but could also suffer from feelings of shame and guilt; alternatively, those involved could be asked to contribute to the preparations and join from the audience
Moderator	Having a skilled moderator can be useful to promote an open atmosphere that is free of shame and blame, as well as contributing to interactivity and time efficiency, and helping to “close the loop” on prior actions
**Process/Content**	
Capturing cases	Some options include case reporting (e.g., an e-mail to a dedicated colleague), using an adverse events registry embedded in the electronic health record system or systematically documenting potential cases during other routine meetings (e.g., clinical handover)
Case selection	A dedicated clinician (e.g., the moderator) can select the cases that will be discussed at M&M, or one could use selection based on specific criteria (e.g., certain severity level, number or type of events). An alternative would be to select all discharged as well as planned cases (this format will be discussed in the paragraph “future directions”). Ensure that topics are relevant to the audience and demonstrate a sense of urgency
Presentations	Fixed presentation formats could be useful to include all relevant aspects of the case as well as other relevant information, such as literature reviews, local/benchmark data as well as system-level and soft/human factors. An option would be to use projected slides and a presentation format followed by a subsequent discussion, but a less formal approach would, for example, be to project the electronic record on a large screen and use a topic list to guide the case discussion 37
Discussions	Pay attention to participant motivation to enhance attendance rates and active participation (e.g., selecting topics of interest, drawing on collective expertise, demonstrating the value of previous M&M-based actions). 19 Stimulate audience participation (e.g., using a moderator), and cultivate an open mindset receptive to input from any participant and all opportunities for improvement
System-level focus	Avoid conclusions such as “be more careful next time” but rather discuss how to support that. Steer discussions to reflection on processes rather than discussing preventability in hindsight. 36
Action plans	Document actions for improvement explicitly (who, what, how, when) and anticipate barriers related to execution (e.g., lack of empowerment). Follow up on previous actions through re-evaluation and feedback. Consider installing a dedicated task force or committee for (supporting) the execution of plans

Abbreviation: M&M, morbidity and mortality conferences.

a Suggestions are based on various publications. 1 5 6 16 19 36 37 38 39 Specific references are added only when deemed useful for readers interested in more detailed descriptions.

### Use of Active Moderator and Structured Formats

Frequently recurring topics in studies on the implementation or optimization of M&M are the importance of strategies aiming to structure the discussion and prevent a too narrow focus on individual performance. The use of a skillful moderator can help to structure as well as deepen discussions and promote a safe atmosphere. 1 4 16 19 29 40 A fixed format could be helpful for presentations and discussions. An example includes a case presentation format that includes a summary of the case with a structured analysis of the complications using a classification system such as Clavien-Dindo, followed by a review of the relevant literature and local as well as published data in relation to this complication. 16 Another example includes reviewing a fixed set of factors per case, such as reason for admission, length of stay, interventions, complications, financial coding, discharge letters, and follow-up visits. 37 Using fixed formats for presentation and discussion, as well as an active role for the moderator could also help to counter the tendency to focus on individual-level factors. Various studies have expressed concerns regarding M&M being too focused on individual performance 11 29 41 42 43 44 45 rather than adopting the systems approach, which has long been advocated for in the safety science and patient safety field. 46 47 Shifting the focus from individual performance to more system-level factors is not only considered essential for achieving sustainable improvements but will likely also help to decrease the risk of shaming and blaming. Cromeens et al describe how restructuring their Paediatric Surgery M&M, using a more structured approach for case analysis, enhanced their ability to identify more system errors. 20 In this publication, the authors acknowledge that they still classified most (79%) of failures as “individual failures,” while quality improvement research has taught us that system errors typically exceed individual errors. However, this was already an improvement since “prior to the implementation of this system nearly all errors identified were individual errors,” and they expect that over time, the identification of systems errors will continue to increase. 20 Other strategies to emphasize a system perspective and widen the focus beyond intraoperative care include discussing a set of similar cases together rather than one individual case. Assessing aggregated local data rather than single cases also decreases the risk of making policy or system changes based on single cases, which are not necessarily representative of the overall local performance. Another approach to create a broader perspective in discussions is to involve all providers involved in the specific care process in a multidisciplinary group discussion. 36

### Use of a Dedicated Committee for Action and Follow-Up

Another frequently highlighted problem in M&M practice is lack of attention for implementation and follow-up of issues identified at the meeting. As described by Sacks et al, all too often it is a case of “rinse and repeat”: the case is discussed, the learning points are highlighted, and then the next presentation begins. 28 A strategy could be to attach a formal quality committee to M&M that could follow up on issues identified at the meeting and ensure that action plans are carried out. 5 This way whatever comes out of M&M can be linked to other quality improvement initiatives in the department or institution, which helps to coordinate actions.

### Attention for Sociological Factors That Are at Play

In addition to more structural factors, factors related to (inter)personal dynamics, such as team spirit and motivation, are often left undiscussed in studies that focus on strategies to optimize M&M. Traditionally, M&M has been instrumental in building professional identities, modelling expected ethical and professional standards. 1 17 It is evident that behaviors and interactions between participants are of great influence on the meeting itself and will likely shape attitudes of trainees. 5 Considering this, it seems useful to dedicate some time to reflect with the local team on what sociological or psychological factors are likely at play in M&M practice, and how to address these. For example, groups with limited experience with M&M may consider starting each conference with explicitly stating the purpose of the meeting as non-blaming, and using a moderator with an active role in discussions, protecting an open and safe atmosphere. To illustrate, the moderator could actively invite comments from participants who may find it difficult to speak up. Also, it may differ per team whether one is familiar and comfortable with multidisciplinary discussions with nurses or colleagues from other departments, which may influence decisions on how to set up M&M meetings (e.g., deciding to first discuss a case in a smaller setting before inviting others, and change this once the group becomes more familiar with this).

## Future Directions for M&M

In our institution, M&M has been carried out in various ways over the years. For a while, the conference was mainly used as means to collectively verify all registered complications in the local database, with only limited time for discussion. At some point, participants considered this approach too focused on administrative requirements, and installed a new format that focused on an in-depth discussion of a single case, supported by evidence from relevant literature. In 2016, this departmental M&M practice was restructured into separate reflective team meetings for each of the surgical subspecialities (e.g., transplant or vascular surgery). Based on advancements in safety science that call for a shift in focus from only cases with a lack of safety to also cases in which safety was successfully created, 48 a new format for the weekly M&M conference was developed: not only complicated cases are discussed, but all last week's discharged cases pass in review. 37 In addition, all cases scheduled for next week are reviewed to anticipate any issues. This way, all (planned) admissions are discussed twice, both in anticipation and in retrospection, which also help to reinforce successful practices such as anticipatory measures that appeared to be effective. These meetings are attended by the entire team of surgeons, residents as well as ward nurses, with providers from other disciplines participating when invited. As a result of discussing all cases, rather than only the complicated ones, this new format appears to provide for a wider focus, thereby creating awareness about the actual ratio between what goes wrong and what goes well. Even the cases with a normal or even better than anticipated clinical course are discussed, which offer an opportunity to reinforce good practices and will moreover stimulate the team's morale. Such a format also seems to help in gaining an understanding of how a team continuously seeks to create high-quality care, which is opposed to evaluating the root cause for a (often rare) event. Future efforts for optimization of M&M could move away from a sole focus on complications, towards making everyday routines more explicit, 49 for example, by discussing a spectrum of cases, irrespective of their (un)complicated outcome. Other avenues for future directions of M&M could find ways to allow patients to participate in the conferences, with the aim of strengthening mutual understanding and gaining insights from another perspective. 39

## Conclusion

Even though M&M practice is a century-old practice in place in many institutions, a golden standard on how to organize and carry out these conferences is lacking. This is also related to the fact that the effectiveness of M&M practice is difficult to measure objectively and quantitatively, hampering comparative studies on different formats. Together with the fact that many sociological factors affect M&M, and specifically the extent of open discussion, this warrants for more qualitative research. Various practical suggestions on how to best execute M&M can be distilled from the literature, such as the use of (i) a skillful and active moderator; (ii) structured formats for case presentation and discussion; and (iii) a dedicated committee to guide improvement plans that ensue from the meeting. By all means, one needs to tailor the format to the local setting. In doing so, it seems important to also reflect on sociological factors that can affect M&M locally, such as how audience composition may influence the extent of open discussion for this particular team. Responding to increasing calls in the safety science field for a wider consideration of also the cases with success rather than only lack thereof, a future direction for M&M would be to use the meeting to broadly reflect on everyday practice by discussing all recently discharged cases rather than only the complicated ones, offering the opportunity to also reinforce successful practices.

## References

[1] Orlander J D Barber T W Fincke B G The morbidity and mortality conference: the delicate nature of learning from error Acad Med 2002 77 10 1001 1006 12377674 10.1097/00001888-200210000-00011

[2] ACGME Program Requirements for Graduate Medical Education in General SurgeryAccessed November 25. 2022, at:https://www.acgme.org/Portals/0/PFAssets/ProgramRequirements/440_general_surgery_2017-07-01.pdf

[3] Kaderbesluit CCMS (Dutch).; 2014. Accessed November 25. 2022, at:http://knmg.artsennet.nl/Opleiding-en-herregistratie/CGS/Regelgeving/Huidigeregelgeving.htm

[4] Gordon L Gordon's Guide to the Surgical Morbidity and Mortality Conference Philadelphia, PA: Hanley & Belfus 1994

[5] Smaggus A Mrkobrada M Marson A Appleton A Effects of efforts to optimise morbidity and mortality rounds to serve contemporary quality improvement and educational goals: a systematic review BMJ Qual Saf 2018 27 01 74 84 10.1136/bmjqs-2017-006632 28971889

[6] Verhagen M J de Vos M S Smaggus A Hamming J F Measuring what matters at morbidity and mortality conferences: a scoping review of effectiveness measures J Patient Saf 2022 18 04 e760 e768 35617601 10.1097/PTS.0000000000000936

[7] Rowland P Cupido N Albert M Kitto S Morbidity and mortality rounds in the medical literature: a text based analysis of shifting knowledge regimes SSM - Qualitative Research in Health 2022 2 100169 10.1016/j.ssmqr.2022.100169

[8] Berenholtz S M Hartsell T L Pronovost P J Learning from defects to enhance morbidity and mortality conferences Am J Med Qual 2009 24 03 192 195 19258468 10.1177/1062860609332370

[9] Aboumatar H J Blackledge C G Jr Dickson C Heitmiller E Freischlag J Pronovost P J A descriptive study of morbidity and mortality conferences and their conformity to medical incident analysis models: results of the morbidity and mortality conference improvement study, phase 1 Am J Med Qual 2007 22 04 232 238 17656727 10.1177/1062860607303292

[10] de Vos M S Healthcare Improvement Based on Learning from Adverse OutcomesPhD thesis. Leiden University Medical Center; 2018. Accessed November 24, 2022, at:https://hdl.handle.net/1887/67419

[11] Xiong X Johnson T Jayaraman D McDonald E G Martel M Barkun A N At the crossroad with morbidity and mortality conferences: lessons learned through a narrative systematic review Can J Gastroenterol Hepatol 2016 2016 7.679196E6 10.1155/2016/7679196 PMC4904689 27446868

[12] Sellier E David-Tchouda S Bal G François P Morbidity and mortality conferences: their place in quality assessments Int J Health Care Qual Assur 2012 25 03 189 196 22755474 10.1108/09526861211210411

[13] Flynn-O'Brien K T Mandell S P Eaton E V Schleyer A M McIntyre L K Surgery and medicine residents' perspectives of morbidity and mortality conference: an interdisciplinary approach to improve ACGME core competency compliance J Surg Educ 2015 72 06 e258 e266 26143516 10.1016/j.jsurg.2015.05.015 PMC12679269

[14] Gore D C National survey of surgical morbidity and mortality conferences Am J Surg 2006 191 05 708 714 16647366 10.1016/j.amjsurg.2006.01.029

[15] Kim M J Fleming F J Peters J H Salloum R M Monson J R Eghbali M E Improvement in educational effectiveness of morbidity and mortality conferences with structured presentation and analysis of complications J Surg Educ 2010 67 06 400 405 21156298 10.1016/j.jsurg.2010.04.005

[16] de Vos M S Marang-van de Mheen P J Smith A D Mou D Whang E E Hamming J F Towards best practices for surgical morbidity & mortality conferences: a mixed methods study J Surg Educ 2018 75 01 33 42 28720425 10.1016/j.jsurg.2017.07.002

[17] Bosk C Forgive and Remember: Managing Medical Failure Chicago, IL: Chicago University Press 1979

[18] Kuper A Nedden N Z Etchells E Shadowitz S Reeves S Teaching and learning in morbidity and mortality rounds: an ethnographic study Med Educ 2010 44 06 559 569 20604852 10.1111/j.1365-2923.2010.03622.x

[19] de Vos M S Hamming J F Marang-van de Mheen P J Barriers and facilitators to learn and improve through morbidity and mortality conferences: a qualitative study BMJ Open 2017 7 11 e018833 10.1136/bmjopen-2017-018833 PMC5695320 29133335

[20] Cromeens B Brilli R Kurtovic K Kenney B Nwomeh B Besner G E Implementation of a pediatric surgical quality improvement (QI)-driven M&M conference J Pediatr Surg 2016 51 01 137 142 26581322 10.1016/j.jpedsurg.2015.10.032

[21] Wasser T Grunschel B D Stevens A Transforming systems of care through a novel resident-led approach to morbidity and mortality conferences Acad Psychiatry 2016 40 06 893 897 27541033 10.1007/s40596-016-0606-z

[22] Gerstein W H Ledford J Cooper J Interdisciplinary quality improvement conference: using a revised morbidity and mortality format to focus on systems-based patient safety issues in a VA hospital: design and outcomes Am J Med Qual 2016 31 02 162 168 25332453 10.1177/1062860614555430

[23] Frey B Doell C Klauwer D The morbidity and mortality conference in pediatric intensive care as a means for improving patient safety Pediatr Crit Care Med 2016 17 01 67 72 26492061 10.1097/PCC.0000000000000550

[24] Carroll J S Edmondson A C Leading organisational learning in health care Qual Saf Health Care 2002 11 01 51 56 12078370 10.1136/qhc.11.1.51 PMC1743554

[25] Dixon-Woods M Leslie M Tarrant C Bion J Explaining Matching Michigan: an ethnographic study of a patient safety program Implement Sci 2013 8 01 70 10.1186/1748-5908-8-70 23786847 PMC3704826

[26] Dixon-Woods M McNicol S Martin G Ten challenges in improving quality in healthcare: lessons from the Health Foundation's programme evaluations and relevant literature BMJ Qual Saf 2012 21 10 876 884 10.1136/bmjqs-2011-000760 PMC3461644 22543475

[27] Orlander J D Fincke B G Morbidity and mortality conference: a survey of academic internal medicine departments J Gen Intern Med 2003 18 08 656 658 12911649 10.1046/j.1525-1497.2003.20824.x PMC1494908

[28] Sacks G D Lawson E H Tillou A Hines O J Morbidity and Mortality Conference 2.0 Ann Surg 2015 262 02 228 229 26164430 10.1097/SLA.0000000000001268

[29] Bechtold M L Scott S Dellsperger K C Hall L W Nelson K Cox K R Educational quality improvement report: outcomes from a revised morbidity and mortality format that emphasised patient safety Postgrad Med J 2008 84 990 211 216 18424579 10.1136/qshc.2006.021139

[30] Kwok E SH Calder L A Barlow-Krelina E Implementation of a structured hospital-wide morbidity and mortality rounds model BMJ Qual Saf 2017 26 06 439 448 10.1136/bmjqs-2016-005459 27358230

[31] Santomauro C M Kalkman C J Dekker S WA Second victims, organizational resilience and the role of hospital administration J Hosp Adm 2014 3 05 95 103

[32] Edmondson A C Learning from failure in health care: frequent opportunities, pervasive barriers Qual Saf Health Care 2004 13 02 ii3 ii9 15576689 10.1136/qshc.2003.009597 PMC1765808

[33] Edmondson A C Lei Z Psychological safety: the history, renaissance, and future of an interpersonal construct Annu Rev Organ Psychol Organ Behav 2014 1 01 23 43

[34] Slater N Sekhon P Bradley N Morbidity and mortality conferences in general surgery: a narrative systematic review Can J Surg 2020 63 03 E211 E222 32386469 10.1503/cjs.009219 PMC7828998

[35] Calder L A Kwok E SH Adam Cwinn A Enhancing the quality of morbidity and mortality rounds: the Ottawa M&M model Acad Emerg Med 2014 21 03 314 321 24628757 10.1111/acem.12330

[36] de Vos M S Hamming J F Marang-van de Mheen P J Learning from morbidity and mortality conferences: focus and sustainability of lessons for patient care J Patient Saf 2021 17 03 231 238 29087979 10.1097/PTS.0000000000000440

[37] Verhagen M J de Vos M S Hamming J F Taking morbidity and mortality conferences to a next level: the resilience engineering concept Ann Surg 2020 272 05 678 683 32889871 10.1097/SLA.0000000000004447

[38] Myren B J de Hullu J A Hermens R PMG Koksma J J Zusterzeel P LM Patient involvement via videoconference at the morbidity and mortality (M&M) meeting during COVID-19 BMJ Open Qual 2022 11 01 e001691 10.1136/bmjoq-2021-001691 PMC8819547 35121576

[39] Myren B J Hermens R PMG Koksma J J Bastiaans S de Hullu J A Zusterzeel P LM Openness to new perspectives created by patient participation at the morbidity and mortality meetingIn: Patient Education and Counseling. Vol. 104.Elsevier Ireland Ltd 2021 343 351 10.1016/j.pec.2020.08.008 33051126

[40] Murayama K M Derossis A M DaRosa D A Sherman H B Fryer J P A critical evaluation of the morbidity and mortality conference Am J Surg 2002 183 03 246 250 11943120 10.1016/s0002-9610(02)00791-2

[41] Bal G Sellier E Tchouda S D François P Improving quality of care and patient safety through morbidity and mortality conferences J Healthc Qual 2014 36 01 29 36 22530618 10.1111/j.1945-1474.2011.00203.x

[42] Hutter M M Rowell K S Devaney L A Identification of surgical complications and deaths: an assessment of the traditional surgical morbidity and mortality conference compared with the American College of Surgeons-National Surgical Quality Improvement Program J Am Coll Surg 2006 203 05 618 624 17084322 10.1016/j.jamcollsurg.2006.07.010

[43] Pierluissi E Fischer M A Campbell A R Landefeld C S Discussion of medical errors in morbidity and mortality conferences JAMA 2003 290 21 2838 2842 14657068 10.1001/jama.290.21.2838

[44] Dimick J B Greenberg C C Understanding gaps in surgical quality: learning to count what cannot be counted Ann Surg 2013 257 01 6 7 23235392 10.1097/SLA.0b013e31827ba13d

[45] Mosher B D Anderson C I Nelson C Kepros J P The significance of nontechnical root causes in morbidity and mortality conference: the delivery of surgical care as a science J Am Coll Surg 2009 209 03 S96 10.1016/j.jamcollsurg.2009.06.240

[46] Vincent C Moorthy K Sarker S K Chang A Darzi A W Systems approaches to surgical quality and safety: from concept to measurement Ann Surg 2004 239 04 475 482 15024308 10.1097/01.sla.0000118753.22830.41 PMC1356252

[47] Dekker S WA Leveson N G The systems approach to medicine: controversy and misconceptions BMJ Qual Saf 2015 24 01 7 9 10.1136/bmjqs-2014-003106 25104796

[48] Hollnagel E Woods D D Leveson N Resilience Engineering: Concepts and Precepts Adelshot, UK: Ashgate 2006

[49] McHugh S K Lawton R O'Hara J K Sheard L Does team reflexivity impact teamwork and communication in interprofessional hospital-based healthcare teams? A systematic review and narrative synthesis BMJ Qual Saf 2020 29 08 672 683 10.1136/bmjqs-2019-009921 PMC7398296 31911544

